# Association of Hydroxylmethyl Glutaryl Coenzyme A Reductase Inhibitors, L-Type Calcium Channel Antagonists, and Biguanides With Rates of Psychiatric Hospitalization and Self-Harm in Individuals With Serious Mental Illness

**DOI:** 10.1001/jamapsychiatry.2018.3907

**Published:** 2019-01-09

**Authors:** Joseph F. Hayes, Andreas Lundin, Susanne Wicks, Glyn Lewis, Ian C. K. Wong, David P. J. Osborn, Christina Dalman

**Affiliations:** 1 Division of Psychiatry, University College London, London, United Kingdom; 2 Department of Public Health Sciences, Epidemiology of Psychiatric Conditions, Substance Use, and Social Environment, Karolinska Institute, Stockholm, Sweden; 3 Centre for Medicines Optimisation Research and Education, Research Department of Practice and Policy, School of Pharmacy, University College London, London, United Kingdom; 4 Department of Pharmacology and Pharmacy, University of Hong Kong, Pokfulam, Hong Kong

## Abstract

**Importance:**

Drug repurposing is potentially cost-effective, low risk, and necessary in psychiatric drug development. The availability of large, routine data sets provides the opportunity to evaluate the potential for currently used medication to benefit people with serious mental illness (SMI).

**Objective:**

To determine whether hydroxylmethyl glutaryl coenzyme A reductase inhibitors (HMG-CoA RIs), L-type calcium channel (LTCC) antagonists, and biguanides are associated with reduced psychiatric hospitalization and self-harm in individuals with SMI.

**Design, Setting, and Participants:**

These within-individual cohort studies of patients with SMI compared rates of psychiatric hospitalization and self-harm during periods of exposure and nonexposure to the study drugs, with adjusting for a number of time-varying covariates. Participants included 142 691 individuals from the entire population of Sweden with a diagnosis of bipolar disorder (BPD), schizophrenia, or nonaffective psychosis (NAP) who were 15 years or older and who were treated with psychiatric medication from October 1, 2005, through December 31, 2016. Data were analyzed from April 1 through August 31, 2018.

**Interventions:**

Treatment with HMG-CoA RIs, LTCC antagonists, or biguanides.

**Main Outcomes and Measures:**

Psychiatric hospitalizations and self-harm admissions.

**Results:**

Among the 142 691 eligible participants, the HMG-CoA RI exposure periods were associated with reduced rates of psychiatric hospitalization in BPD (adjusted hazard ratio [aHR], 0.86; 95% CI, 0.83-0.89; *P* < .001), schizophrenia (aHR, 0.75; 95% CI, 0.71-0.79; *P* < .001), and NAP (aHR, 0.80; 95% CI, 0.75-0.85; *P* < .001) and reduced self-harm rates in BPD (aHR, 0.76; 95% CI, 0.66-0.86; *P* < .001) and schizophrenia (aHR, 0.58; 95% CI, 0.45-0.74; *P* < .001). Exposure to LTCC antagonists was associated with reduced rates of psychiatric hospitalization and self-harm in subgroups with BPD (aHRs, 0.92 [95% CI, 0.88-0.96; *P* < .001] and 0.81 [95% CI, 0.68-0.95; *P* = .01], respectively), schizophrenia (aHRs, 0.80 [95% CI, 0.74-0.85; *P* < .001] and 0.30 [95% CI, 0.18-0.48; *P* < .001], respectively), and NAP (aHRs, 0.89 [95% CI, 0.83-0.96; *P* = .002] and 0.56 [95% CI, 0.42-0.74; *P* < .001], respectively). During biguanide exposure, psychiatric hospitalization rates were reduced in subgroups with BPD (aHR, 0.80; 95% CI, 0.77-0.84; *P* < .001), schizophrenia (aHR, 0.73; 95% CI, 0.69-0.77; *P* < .001), and NAP (aHR, 0.85; 95% CI, 0.79-0.92; *P* < .001), and self-harm was reduced in BPD (aHR, 0.73; 95% CI, 0.62-0.84; *P* < .001) and schizophrenia (aHR, 0.64; 95% CI, 0.48-0.85; *P* < .001).

**Conclusions and Relevance:**

This study provides additional evidence that exposure to HMG-CoA RIs, LTCC antagonists, and biguanides might lead to improved outcomes for individuals with SMI. Given the well-known adverse event profiles of these agents, they should be further investigated as repurposed agents for psychiatric symptoms.

## Key Points

**Question:**

Are drugs in common use for physical health problems (hydroxylmethyl glutaryl coenzyme A reductase inhibitors, L-type calcium channel antagonists, and biguanides) associated with reduced rates of psychiatric hospitalization and self-harm in individuals with serious mental illness?

**Findings:**

In this series of within-individual cohort studies of 142 691 patients with bipolar disorder, schizophrenia, or nonaffective psychosis, exposure to any of the study drugs was associated with reduced rates of psychiatric hospitalizaiton compared with unexposed periods. Self-harm was reduced in patients with bipolar disorder and schizophrenia during exposure to all study drugs and in patients with nonaffective psychosis taking L-type calcium channel antagonists.

**Meaning:**

Hydroxylmethyl glutaryl coenzyme A reductase inhibitors, L-type calcium channel antagonists, and biguanides hold potential as repurposed agents in serious mental illness, and the central nervous system mechanism of action of these drugs requires further investigation.

## Teaser

This series of within-individual cohort studies assesses whether hydroxylmethyl glutaryl coenzyme A (HMG-CoA) reductase inhibitors, L-type calcium channel antagonists, and biguanides are associated with reduced rates of psychiatric hospitalization and self-harm in individuals with serious mental illness.

## Introduction

Serious mental illnesses (SMIs), including bipolar disorder (BPD), schizophrenia, and nonaffective psychoses (NAP), are associated with high levels of morbidity and are challenging to treat. Many drugs have been identified as having potential for repurposing in these disorders.^1,2^ We cross-referenced these drugs with the most commonly prescribed drugs in the general population.^3^ We chose to investigate the following 3 classes of drugs for which we might expect clinical benefit: hydroxylmethyl glutaryl coenzyme A reductase inhibitors (HMG-CoA RIs; ie, statins), L-type calcium channel (LTCC) antagonists (eg, verapamil hydrochloride), and biguanides (eg, metformin hydrochloride).

A recent meta-analysis of statins as adjunctive therapy for schizophrenia included 6 placebo-controlled randomized clinical trials (RCTs) and found a reduction in the Positive and Negative Symptom Scale scores in patients receiving statins for 12 weeks; however, only 169 received active treatment, and the reduction fell below the threshold considered clinically meaningful.^4^ Theorized mechanisms for HMG-CoA RI psychiatric action are via anti-inflammatory effects or via increased absorption and central nervous system uptake of antipsychotics.^4^ A systematic review and meta-analysis of LTCC antagonists for the treatment of BPD^5^ included 2 RCTs of verapamil vs placebo and 4 of verapamil vs lithium for the treatment of mania. The investigators found no evidence of an effect. However, no trials of other LTCC antagonists and no studies in BPD depression or prophylaxis have been performed. Evidence of calcium dysregulation in BPD has long been available; this evidence is supported by more recent pharmacologic, genetic, and molecular findings.^6^ Similarly, calcium signaling is implicated in the etiology of schizophrenia, where LTCC antagonists are also a potential adjuvant treatment.^7^ However, very limited and inconsistent trial evidence for their use in this disorder exists.^8^ A trial of metformin has been undertaken to counter antipsychotic-related weight gain,^9^ but metformin is also hypothesized to improve cognitive and mood dysfunction symptoms via mitigation of metabolic disturbances.^10^ Therefore, although none of these drugs have been comprehensively investigated as repurposed agents to improve mental disorders, each has a theoretical basis for effectiveness.

We investigated whether people in the Swedish population with SMI had lower rates of psychiatric hospitalization and self-harm during periods when they were prescribed HMG-CoA RIs, LTCC antagonists, and biguanides. Owing to the risks of confounding by indication, we used a design that compared within-individual periods with and without medication exposure,^11^ which controls for time-fixed confounders. In view of the proposed mechanism of action of each drug on psychiatric symptoms, we hypothesized that effects would be similar across SMI subtypes. We also examined rates of nonpsychiatric hospitalization in these groups and hypothesized that, contrary to psychiatric effects, nonpsychiatric hospitalizations would only be reduced in the group exposed to biguanides because these agents have an acute physical effect, whereas HMG-CoA RIs and LTCC antagonists have a longer-term mechanism of action.

## Methods

### Study Population

We collected data from national registers of Sweden from January 1, 1973, until December 31, 2016. For the purpose of this study, the Total Population Register, Migration Register, Cause of Death Register, Prescribed Drug Register, and National Patient Registers were linked. The Prescribed Drug Register contains data on all prescriptions collected from July 1, 2005, onward (excluding over-the-counter medications and drugs used in hospitals).^3^ The National Patient Registers contain inpatient records from 1964 onward and outpatient records from 2001 onward. These registers contain sociodemographic and medical information on each resident of Sweden. Ethical approval for the study was obtained via the Regional Ethical Review Board in Stockholm, Sweden, which waived the need for informed consent.

### Definition of SMI

We defined diagnoses of BPD, schizophrenia, and NAP in line with previous research using Swedish National Patient Registers.^12,13^ Individuals had at least 1 record of inpatient or outpatient contact with codes from the *International Classification of Diseases, Ninth Revision* (*ICD-9*) and *International Statistical Classification of Diseases and Related Health Problems, Tenth Revision* (*ICD-10*) consistent with the diagnosis (eTable 1 in the Supplement) and a period of drug treatment with an appropriate psychotropic medication (antipsychotic, lithium, or anticonvulsant class mood stabilizer) from October 1, 2005, through December 31, 2016. Validation studies suggest that using inpatient SMI diagnosis alone has high positive predictive power (0.94) but potentially misses suitable patients owing to type II error (negative predictive power, 0.23).^14^

### Exposure to Study Medications

The main exposure was defined as treatment with HMG-CoA RIs, LTCC antagonists, or biguanides (eTable 2 in the Supplement) in the Prescribed Drug Register. In routine practice in Sweden, oral medications are rarely dispensed for longer than 3 months at a time. In line with previous studies, we defined a medication period as a sequence of at least 2 prescriptions, with no more than 3 months (93 days) between any 2 consecutive prescriptions.^15,16^ Therefore, individuals were defined as exposed to medication during the interval between 2 dispensed prescriptions unless the dispensed prescriptions occurred more than 3 months apart. To determine whether an individual was exposed or nonexposed to the medication initially, the follow-up start was set to October 1, 2005, because the coverage of the Prescribed Drug Register started on July 1, 2005.

### Outcomes

Outcomes were psychiatric hospitalization (identified by ward admission codes and *ICD-9* and *ICD-10* codes for admission indication) and self-harm (with suicidal and undetermined intent) (eTable 1 in the Supplement). We also examined rates of nonpsychiatric hospitalization. Outcome data came from the National Patient Register.

### Time-Varying Confounders

As a result of the within-individual design, time-fixed covariates (eg, sex, age at illness onset) cannot confound any association between the study drug and the outcome of interest. Time-varying confounders were included to capture the potential for changes in psychiatric symptoms over time, including age, year, number of previous outcome events (ie, hospitalizations, episodes of self-harm), exposure to psychiatric medication (ie, mood stabilizer or antipsychotic), and a count of exposed and nonexposed periods.

### Statistical Analysis

Data were analyzed from April 1 through August 31, 2018. We performed within-individual analyses using stratified Cox proportional hazards regression. This method, by design, exclusively draws information from individuals who ever experienced the outcome of interest during follow-up.^17^ Therefore, a different cohort is identified and analyzed for each combination of drug exposure and outcome. We split follow-up time into consecutive periods. A new period started after a medication switch (ie, starting and stopping medication) or an outcome event (after the date of discharge). For the latter, we restarted the period at baseline (ie, the time since the last event). Drug treatments were defined as time-varying dichotomous exposures. We estimated adjusted hazard ratios (aHRs) and 95% CIs for differences in outcome event rate between medication exposure and nonexposure periods; 2-sided *P* values were calculated using Wald tests.

### Additional Analyses

We tested for an interaction between HMG-CoA RI and antipsychotic medication exposure, because of the theory that HMG-CoA RIs may increase uptake of antipsychotics by the central nervous system.^4^ We then performed several sensitivity analyses. First, we examined differences in rates of the 3 outcomes in periods that patients with SMI were exposed or not exposed to thiazide diuretics. We chose this class of drug as a negative control; no evidence suggests that exposure to thiazide diuretics should improve psychiatric outcomes, so lower rates during exposed periods might suggest that stability of SMI symptoms is associated with a period of medication adherence rather than a direct effect of the medication on psychiatric symptoms (ie, reverse causality). Second, we adjusted for time with and without each of the other study drugs and physical health problems experienced by the individual (cardiovascular or cerebrovascular disease, type 2 diabetes, hypertension, and hyperlipidemia). Third, we excluded from the analysis individuals who died during the follow-up period because they may have a more rapidly varying risk of the outcomes of interest. Fourth, we excluded psychiatric admissions, which were prompted by admissions for self-harm (ie, a psychiatric admission in the 7 days after a self-harm admission), to examine whether self-harm and psychiatric admissions were independent. Finally, we extended the definition of the drug exposure period and defined it as constant if a new prescription was issued within 4 months (124 days) of the previous prescription and extended the exposure period to 2 months (62 days) after the last dispensed date to account for potential exposure misclassification in our primary analysis.

## Results

These studies drew from the entire population of Sweden who ever received an inpatient or outpatient diagnosis of SMI and were treated with an antipsychotic or mood stabilizer medication from October 1, 2005, through December 31, 2016 (eFigure in the Supplement). This base population included 76 759 patients with BPD, 30 954 with schizophrenia, and 34 978 with NAP for a total of 142 691 patients.

### HMG-CoA Reductase Inhibitors

Of the patients described above, 6176 with BPD (3493 female [56.6%] and 2683 male [43.4%]; mean [SD] age, 55.3 [12.3] years), 2705 with schizophrenia (1084 female [40.1%] and 1621 male [59.9%]; mean [SD] age, 49.6 [12.6] years), and 2958 with NAP (1603 female [54.2%] and 1355 male [45.8%]; mean [SD] age, 57.8 [15.0] years) were also prescribed HMG-CoA RIs and experienced psychiatric hospitalization (Table 1). All subgroups of SMI had reduced rates of psychiatric hospitalization during HMG-CoA RI exposure after adjusting for age, year, number of previous hospitalizations, psychiatric medication exposure, and number of exposure periods (aHR for BPD, 0.86 [95% CI, 0.83-0.89]; aHR for schizophrenia, 0.75 [95% CI, 0.71-0.79]; and aHR for NAP, 0.80 [95% CI, 0.75-0.85]; *P* < .001 for all). Individuals with BPD (aHR, 0.76; 95% CI, 0.66-0.86; *P* < .001) or schizophrenia diagnoses (aHR, 0.58; 95% CI, 0.45-0.74; *P* < .001) had lower rates of self-harm during HMG-CoA RI exposure periods compared with nonexposure periods (Table 2; eTable 3 in the Supplement). Exposure status for HMG-CoA RIs was not associated with nonpsychiatric hospitalization, apart from in the NAP subgroup (aHR, 1.04; 95% CI, 1.01-1.08; *P* = .02) (Table 2; eTable 4 in the Supplement). We found no evidence of an interaction between HMG-CoA RI and antipsychotic exposure. Adjustment for additional time-varying confounders, such as LTCC antagonist and biguanide exposure, cardiovascular or cerebrovascular disease, type 2 diabetes, hypertension, and hyperlipidemia, did not affect the results for psychiatric hospitalization or self-harm outcomes, but nonpsychiatric hospitalization rates were significantly reduced during HMG-CoA RI exposure periods (aHR, 0.73; 95% CI, 0.72-0.74; *P* < .001) (Table 3). Excluding patients who died during follow-up did not change results for rates of psychiatric hospitalization or self-harm, but HMG-CoA RI exposure periods became associated with increased rates of nonpsychiatric hospitalization (aHR, 1.08; 95% CI, 1.06-1.11; *P* < .001). After excluding psychiatric hospitalizations prompted by self-harm, the association between HMG-CoA RI exposure periods and psychiatric hospitalization remained similar (aHR, 0.82; 95% CI, 0.80-0.84; *P* < .001). Redefining exposure period as constant for less than 4 months between prescriptions and extending to 2 months after the last prescription date did not change the findings.

**Table 1.** Baseline Characteristics of Patients Experiencing Psychiatric Hospitalization

Characteristic	Drug Exposure by SMI Subgroup								
	HMG-CoA Reductase Inhibitors			LTCC Antagonists			Biguanides		
	BPD (n = 6176)	Schizophrenia (n = 2705)	NAP (n = 2958)	BPD (n = 4636)	Schizophrenia (n = 1581)	NAP (n = 2337)	BPD (n = 3493)	Schizophrenia (n = 2294)	NAP (n = 1562)
Age at start of follow-up, mean (SD), y	55.3 (12.3)	49.6 (12.6)	57.8 (15.0)	56.1 (13.5)	54.2 (12.1)	60.9 (15.4)	50.5 (14.4)	47.2 (13.2)	50.5 (16.4)
Female, No. (%)	3493 (56.6)	1084 (40.1)	1603 (54.2)	2676 (57.7)	701 (44.3)	1406 (60.2)	1998 (57.2)	974 (42.4)	839 (53.7)
Swedish born, No. (%)	5250 (85.0)	2013 (74.4)	2225 (75.2)	3990 (86.1)	1217 (77.0)	1802 (77.1)	2883 (82.5)	1652 (72.0)	1075 (68.8)
Died during follow-up, No. (%)	1208 (19.6)	484 (17.9)	763 (25.8)	985 (21.2)	410 (25.9)	731 (31.3)	629 (18.0)	417 (18.2)	316 (20.2)
Psychiatric treatment during follow-up, No. (%)									
Antipsychotic	5458 (88.4)	2695 (99.6)	2883 (97.5)	3968 (85.6)	1572 (99.4)	2274 (97.3)	3209 (91.9)	2291 (99.9)	1528 (97.8)
Lithium	2900 (47.0)	145 (5.4)	96 (3.2)	2240 (48.3)	74 (4.7)	65 (2.8)	1633 (46.75)	138 (6.0)	85 (5.4)
Anticonvulsant	3511 (56.8)	568 (21.0)	474 (16.0)	2634 (56.8)	319 (20.2)	309 (13.2)	1998 (57.2)	488 (21.3)	275 (17.6)
Physical health by end of follow-up, No. (%)									
Cardiovascular or cerebrovascular disease	4546 (73.6)	1591 (58.8)	2237 (75.6)	3800 (82.0)	1179 (74.6)	1968 (84.2)	2137 (61.2)	1128 (49.2)	957 (61.3)
Type 2 diabetes	2418 (39.2)	1281 (47.4)	1068 (36.1)	1228 (26.5)	539 (34.1)	611 (26.1)	2659 (76.1)	1680 (73.2)	1177 (75.4)
Hypertension	3237 (52.4)	1002 (37.0)	1650 (55.8)	3376 (72.8)	1029 (65.1)	1739 (74.4)	1593 (45.6)	750 (32.7)	727 (46.5)
Hyperlipidemia	1207 (19.5)	396 (14.6)	643 (21.7)	518 (11.2)	128 (8.1)	327 (14.0)	461 (13.2)	213 (9.3)	213 (13.4)
No. of study drug exposure periods, median (IQR)	5 (3-11)	3 (3-7)	5 (3-11)	5 (3-9)	3 (3-7)	5 (3-10)	5 (3-9)	4 (3-7)	5 (3-9)
Rate of admission per 100 person-years (95% CI)	46.38 (45.86-46.90)	46.30 (45.51-47.10)	32.82 (32.19-33.47)	42.99 (42.41-43.58)	45.46 (44.43-46.51)	31.42 (30.71-32.15)	55.94 (55.18-56.71)	48.46 (47.58-49.35)	41.80 (40.82-42.81)
No. of psychiatric admissions during exposure/nonexposure to study drug	7432/22 649	3714/9395	2002/7947	4002/16 881	1707/5617	1429/5940	4948/15 548	3361/8200	1402/5360
Time of exposure/nonexposure to study drug, person-years	1.6 × 10^4^/4.9 × 10^4^	9.0 × 10^3^/1.9 × 10^4^	7.1 × 10^3^/2.3 × 10^4^	9.6 × 10^3^/3.8 × 10^4^	4.2 × 10^3^/1.1 × 10^4^	4.7 × 10^3^/1.8 × 10^4^	9.8 × 10^3^/2.7 × 10^4^	8.1 × 10^3^/1.6 × 10^4^	3.8 × 10^3^/1.2 × 10^4^

Abbreviations: BPD, bipolar disorder; HMG-CoA, hydroxylmethyl glutaryl coenzyme A; IQR, interquartile range; LTCC, L-type calcium channel; NAP, nonaffective psychosis; SMI, serious mental illness.

**Table 2.** Psychiatric Hospitalization, Self-harm, and Nonpsychiatric Hospitalization During Drug Exposure vs Nonexposure Periods

SMI	Outcome, HR (95% CI)					
	Psychiatric Hospitalization		Self-harm		Nonpsychiatric Hospitalization	
	Unadjusted	Adjusted^a^	Unadjusted	Adjusted^a^	Unadjusted	Adjusted^a^
**HMG-CoA Reductase Inhibitors**						
BPD	0.81 (0.79-0.84)	0.86 (0.83-0.89)	0.76 (0.67-0.85)	0.76 (0.66-0.86)	1.01 (0.98-1.03)	1.00 (0.98-1.03)
*P* value	<.001	<.001	<.001	<.001	.64	.49
Schizophrenia	0.74 (0.71-0.78)	0.75 (0.71-0.79)	0.64 (0.51-0.81)	0.58 (0.45-0.74)	1.08 (1.03-1.12)	1.03 (0.99-1.08)
*P* value	<.001	<.001	<.001	<.001	<.001	.13
NAP	0.79 (0.75-0.83)	0.80 (0.75-0.85)	0.93 (0.78-1.12)	0.86 (0.71-1.05)	0.98 (0.94-1.01)	1.04 (1.01-1.08)
*P* value	<.001	<.001	.45	.15	.13	.02
**LTCC Antagonists**						
BPD	0.82 (0.79-0.85)	0.92 (0.88-0.96)	0.81 (0.70-0.95)	0.81 (0.68-0.95)	1.03 (1.00-1.06)	1.01 (0.98-1.04)
*P* value	<.001	<.001	.007	.01	.01	.66
Schizophrenia	0.78 (0.73-0.83)	0.80 (0.74-0.85)	0.49 (0.32-0.77)	0.30 (0.18-0.48)	1.04 (1.00-1.10)	1.03 (0.98-1.08)
*P* value	<.001	<.001	.002	<.001	.08	.20
NAP	0.78 (0.73-0.83)	0.89 (0.83-0.96)	0.81 (0.63-1.04)	0.56 (0.42-0.74)	0.96 (0.92-0.99)	0.97 (0.93-1.01)
*P* value	<.001	.002	.10	<.001	.02	.13
**Biguanides**						
BPD	0.69 (0.67-0.72)	0.80 (0.77-0.84)	0.74 (0.65-0.84)	0.73 (0.62-0.84)	0.86 (0.83-0.90)	0.84 (0.81-0.87)
*P* value	<.001	<.001	<.001	<.001	<.001	<.001
Schizophrenia	0.59 (0.56-0.63)	0.73 (0.69-0.77)	0.70 (0.55-0.90)	0.64 (0.48-0.85)	0.91 (0.87-0.96)	0.84 (0.80-0.89)
*P* value	<.001	<.001	.005	<.001	<.001	<.001
NAP	0.66 (0.61-0.71)	0.85 (0.79-0.92)	0.82 (0.66-1.02)	0.91 (0.71-1.16)	0.85 (0.80-0.89)	0.85 (0.80-0.90)
*P* value	<.001	<.001	.08	.44	<.001	<.001

Abbreviations: BPD, bipolar disorder; HMG-CoA, hydroxylmethyl glutaryl coenzyme A; HR, hazard ratio; LTCC, L-type calcium channel; NAP, nonaffective psychosis; SMI, serious mental illness.

^a^ Adjusted for time-varying covariates, including age, year, number of previous outcome events, and psychiatric medication exposure (antipsychotic, lithium, or anticonvulsant mood stabilizer).

**Table 3.** Additional Analyses of Psychiatric Hospitalization, Self-harm, and Nonpsychiatric Hospitalization During Drug Exposure vs Nonexposure Periods

Study Drug	Outcome, HR (95% CI)^**a**^		
	Psychiatric Hospitalization	Self-harm	Nonpsychiatric Hospitalization
Negative control			
Thiazide diuretics	0.99 (0.93-1.05)	0.94 (0.75-1.19)	0.92 (0.88-0.96)
*P* value	.64	.61	<.001
**Additionally Adjusted for Other Study Drugs and Physical Health Problems**^b^			
HMG-CoA reductase inhibitors	0.81 (0.79-0.84)	0.75 (0.67-0.83)	0.73 (0.72-0.74)
*P* value	<.001	<.001	<.001
LTCC antagonists	0.87 (0.84-0.90)	0.69 (0.60-0.79)	0.95 (0.93-0.97)
*P* value	<.001	<.001	<.001
Biguanides	0.68 (0.66-0.71)	0.72 (0.63-0.82)	0.76 (0.74-0.78)
*P* value	<.001	<.001	<.001
**Excluding Individuals Who Died During Follow-up**			
HMG-CoA reductase inhibitors	0.83 (0.80-0.85)	0.75 (0.67-0.85)	1.08 (1.06-1.11)
*P* value	<.001	<.001	<.001
LTCC antagonists	0.89 (0.86-0.92)	0.75 (0.64-0.88)	1.09 (1.06-1.12)
*P* value	<.001	<.001	<.001
Biguanides	0.77 (0.75-0.80)	0.73 (9.64-0.83)	0.91 (0.88-0.94)
*P* value	<.001	<.001	<.001
**Exposure Is Constant if 4 mo Between Prescriptions and Ends 2 mo After Final Prescription**			
HMG-CoA reductase inhibitors	0.80 (0.78-0.82)	0.76 (0.69-0.84)	1.00 (0.99-1.02)
*P* value	<.001	<.001	.71
LTCC antagonists	0.91 (0.88-0.94)	0.69 (0.61-0.79)	1.02 (1.00-1.04)
*P* value	<.001	<.001	.02
Biguanides	0.83 (0.81-0.86)	0.74 (0.66-0.83)	0.93 (0.91-0.95)
*P* value	<.001	<.001	<.001

Abbreviations: BPD, bipolar disorder; HMG-CoA, hydroxylmethyl glutaryl coenzyme A; HR, hazard ratio; LTCC, L-type calcium channel; NAP, nonaffective psychosis.

^a^ Adjusted for time-varying covariates, including age, year, number of previous outcome events, and psychiatric medication exposure (antipsychotic, lithium, and anticonvulsant mood stabilizer).

^b^ Additionally adjusted for calcium channel antagonist, HMG-CoA reductase inhibitor, and biguanides exposure periods, cardiovascular or cerebrovascular disease diagnosis, type 2 diabetes mellitus, hypertension, and hyperlipidemia.

### LTCC Antagonists

Of those meeting inclusion criteria and prescribed LTCC antagonists, 4636 with BPD (2676 female [57.7%] and 1960 male [42.3%]; mean [SD] age, 56.1 [13.5] years), 1581 with schizophrenia (701 female [44.3%] and 880 male [55.7%]; mean [SD] age, 54.2 [12.1] years), and 2337 with NAP (1406 female [60.2%] and 931 male [39.8%]; mean [SD] age, 60.9 [15.4] years) experienced psychiatric hospitalization (Table 1). During periods of LTCC antagonist exposure, all groups had reduced psychiatric admissions (aHR for BPD, 0.92 [95% CI, 0.88-0.96; *P* < .001]; aHR for schizophrenia, 0.80 [95% CI, 0.74-0.85; *P* < .001]; and aHR for NAP, 0.89 [95% CI, 0.83-0.96; *P* = .002]) and reduced self-harm events (aHR for BPD, 0.81 [95% CI, 0.68-0.95; *P* = .01]; aHR for schizophrenia, 0.30 [95% CI, 0.18-0.48; *P* < .001]; aHR for NAP, 0.56 [95% CI, 0.42-0.74; *P* < .001]) (Table 2 and eTable 3 in the Supplement). Exposure to LTCC antagonists was not associated with the nonpsychiatric hospitalization rate (eTable 4 in the Supplement). Additional adjustment for the other study drugs and physical health problems did not affect the psychiatric hospitalization or self-harm results, but nonpsychiatric admissions were significantly reduced during LTCC antagonist exposure (aHR, 0.95; 95% CI, 0.93-0.97; *P* < .001) (Table 3). Excluding patients who died suggested an increased rate of nonpsychiatric admission during LTCC antagonist exposure (aHR, 1.09; 95% CI, 1.06-1.12; *P* < .001). Excluding psychiatric admissions prompted by self-harm did not alter the association (aHR for all SMI, 0.86; 95% CI, 0.83-0.88; *P* < .001). Redefining the drug exposure period did not alter our findings.

### Biguanides

In total, 7349 patients were exposed to metformin and had psychiatric hospitalizations during follow-up, of whom 3493 had BPD (1998 female [57.2%] and 1495 male [42.8%]; mean [SD] age, 50.5 [14.4] years), 2294 had schizophrenia (974 female [42.4%] and 1320 male [57.5%]; mean [SD] age, 47.2 [13.2] years), and 1562 had NAP (839 female [53.7%] and 723 male [46.3%]; mean [SD] age, 50.5 [16.4] years) (Table 1). All subgroups had similarly reduced psychiatric hospitalization rates during metformin exposed compared with unexposed periods (aHR for BPD, 0.80 [95% CI, 0.77-0.84; *P* < .001]; aHR for schizophrenia, 0.73 [95% CI, 0.69-0.77; *P* < .001]; and aHR for NAP, 0.85 [95% CI, 0.79-0.92; *P* < .001]). Self-harm was reduced in the BPD (aHR, 0.73; 95% CI, 0.62-0.84; *P* < .001) and schizophrenia (aHR, 0.64; 95% CI, 0.48-0.85; *P* < .001) subgroups (Table 2 and eTable 3 in the Supplement). Across all diagnostic groups, metformin exposure was associated with reduced rates of nonpsychiatric hospitalization (aHR for BPD, 0.84 [95% CI, 0.81-0.87]; aHR for schizophrenia, 0.84 [95% CI, 0.80-0.89]; and aHR for NAP, 0.85 [95% CI, 0.80-0.90]; *P* < .001 for all) (eTable 4 in the Supplement). Additional adjustment for the other study drugs and physical health problems, exclusion of those who died during follow-up, and redefining the exposure period did not affect the results (Table 3), nor did exclusion of psychiatric admissions in the 7 days after self-harm (aHR, 0.79; 95% CI, 0.77-0.81; *P* < .001).

### Thiazide Diuretics

No evidence suggested that thiazide diuretic exposure was associated with psychiatric hospitalization (aHR, 0.99; 95% CI, 0.93-1.05; *P* = .64) or self-harm (aHR, 0.94; 95% CI, 0.75-1.19; *P* = .61). However, exposure was associated with a reduced rate of nonpsychiatric admissions (aHR, 0.92; 95% CI, 0.88-0.96; *P* < .001) (Table 3 and eTable 5 in the Supplement).

## Discussion

As far as we are aware, this study is the first to suggest that periods of exposure to HMG-CoA RIs, LTCC antagonists, and biguanides are associated with lower rates of psychiatric admission and self-harm in patients with SMI. Each of these drugs has a theoretical basis for effectively reducing psychiatric symptoms.

We found that periods of HMG-CoA RI exposure were associated with reduced psychiatric hospitalization in all subgroups of SMI and with reduced self-harm in BPD and schizophrenia. Several actions of HMG-CoA RIs could explain our findings. Statins are anti-inflammatory, with effects on levels of interleukin-1β, interleukin-6, tumor necrosis factor, and C-reactive protein.^18,19^ Extensive evidence suggests that systemic and neuroinflammatory processes are involved in the pathophysiology of psychiatric disorders.^4^ Alternatively (or synergistically), HMG-CoA RIs may interact with other substrates for P-glycoprotein (including antipsychotic medications). We found no evidence of interaction between periods of statin and antipsychotic exposure in our study. However, to assess this mechanism, we would need to specifically examine drugs with a high affinity for P-glycoprotein, such as quetiapine fumarate or risperidone.^20^ The low number of events in those exposed to specific antipsychotics prevented robust analysis. In addition, animal models have found that statins up-regulate *N*-methyl-d-aspartate receptors^21^ and increase muscarinic receptor binding,^22^ potentially producing neuroprotective effects, and increase dopamine receptor levels centrally,^23^ potentially causing antipsychotic effects. Any of these mechanisms may reflect the relatively acute effect of statins on rates of psychiatric hospitalization and self-harm that we observe. In contrast, the mechanism for treatment of hypercholesterolemia is slower, involving inhibition of HMG-CoA reductase, a resultant reduction in hepatocyte cholesterol level, and an increase of hepatic low-density lipoprotein receptor levels, which clear circulating low-density lipoprotein and its precursors.^24^

Exposure to LTCC antagonists was associated with reduced rates of psychiatric hospitalization and self-harm. The LTCCs have been implicated in the physiopathology of a range of mental health conditions, including mood disorders, addiction, dementia, sleep disorders,^6^ and psychotic illness.^7^ Inhibition of LTCCs has cardiodepressant and vasodilatory actions, but isoforms of LTCCs are widely distributed beyond the cardiovascular system, including neurons.^25^ These isoforms appear to control emotional behavior in animal models and are associated with antidepressant and anxiolytic effects.^25^

Periods of metformin exposure were associated with reduced psychiatric and nonpsychiatric hospitalization across all SMI subgroups. Self-harm was reduced in individuals with BPD and schizophrenia during biguanide exposure. Metformin has been widely investigated in psychiatry because of its potential to attenuate antipsychotic-induced weight gain, including in patients without diabetes.^26^ Unfortunately, these trials did not routinely report changes in psychiatric symptoms. Metformin may potentially improve symptoms in SMI because of underlying disturbed cerebral use of glucose, particularly in brain areas linked to cognitive impairments in schizophrenia.^27^

Hypercholesterolemia, hypertension, diabetes, and prediabetes are more common in people with SMI, and evidence suggests that they are not as rigorously treated as in the general population.^28^ Our findings notwithstanding, more effort should be made to prescribe these drugs when individuals with SMI fulfill established clinical criteria for their use. In addition, use of these medications within clinical guidelines is already broader than in the general population. For example, a statin will be indicated in any man older than 60 years with SMI, but no other risk factors for cardiovascular disease, according to risk calculators,^29,30^ and adjunctive metformin therapy is recommended to manage antipsychotic-associated weight gain.^28^ All 3 classes have relatively good adverse event profiles.^31,32,33^ Ideally, further RCTs of these drugs will be conducted that are appropriately powered, with meaningful clinical end points.

### Strengths and Limitations

Compared with possible RCTs, our large, population-based longitudinal sample avoids selection bias because it potentially includes all individuals. In RCTs, recruiting representative samples, such as actively suicidal participants or those who are acutely unwell, is more challenging. Our study is therefore potentially more generalizable and representative. However, to be included in the study, individuals had to be prescribed a study drug, suggesting poor physical health, and experience at least 1 outcome, suggesting poor mental health. We therefore potentially included the most unwell individuals. In addition, most individuals were older than 50 years at the start of follow-up, so survival bias is possible. Whether our results generalize to the wider population with SMI remains unclear, but the group studied would usually be considered most difficult to treat. The comparison of exposed and unexposed periods within the same individual automatically controls for time-invariant confounders, reducing the likelihood of confounding by indication. We then additionally adjusted for potentially important time-varying confounders. However, as with other observational designs, we cannot rule out residual and unmeasured time-varying confounding. In particular, confounding may be linked to varying severity of SMI symptoms. We attempted to investigate whether periods of symptom stability were associated with adherence to any prescribed medication and a reduced rate of psychiatric hospitalization and self-harm by examining nonpsychiatric hospitalization rates and by studying a drug class that should have no effect on psychiatric outcomes. Nonpsychiatric hospitalization was not associated with HMG-CoA RI or LTCC antagonist exposure periods, suggesting no global effect on health service use. The effects seem specific to psychiatric outcomes. Biguanide exposure was probably associated with reduced nonpsychiatric hospitalization because this class of medication has more acute effects. Thiazide diuretics, a group of medications that have no theorized basis for improving psychiatric symptoms, were not associated with rates of psychiatric hospitalization or self-harm rate but were associated with reduced rates of nonpsychiatric hospitalization. This finding suggests that medication adherence in and of itself is not a proxy for mental state stability, which would be reflected in reduced rates of psychiatric hospitalization and self-harm.

Exposure misclassification is a potential risk; in our primary analysis, patients were considered unexposed after the date of their last prescription. This classification potentially underestimates exposure time but would bias estimates toward the null. In the sensitivity analysis with increased exposure time, effect estimates were comparable. Unfortunately, the Prescribed Drug Register does not include information on dose and days supply. Similarly, we lack direct information on treatment adherence and defined patients collecting 2 or more prescriptions as adherent. Nonadherence would mean our reported associations represent underestimates of the true effect. Misclassification of SMI subgroup is possible, but given the inclusion criteria for this study, we were unlikely to include patients who do not have some form of SMI.^14,34,35^ Self-harm that did not result in hospital attendance is not captured by our study.

## Conclusions

If substantiated, this study has considerable implications for clinical practice and drug development. The study drugs—HMG-CoA RIs, LTCC antagonists, and biguanides—are globally licensed, commonly used, cheap, and relatively safe medications. They are therefore ideal candidates for repurposing. Understanding their mode of action on the central nervous system may facilitate better understanding of the pathophysiology of SMI and offer opportunities for innovative pharmacotherapy development.

## Notes

Supplement. **eFigure.** Patient Flow Diagram **eTable 1.** *ICD* Diagnostic Codes **eTable 2.** Anatomical Therapeutic Chemical Classification System Codes **eTable 3.** Baseline Characteristics of Patients Treated for Self-Harm **eTable 4.** Baseline Characteristics of Patients Experiencing Nonpsychiatric Hospitalization **eTable 5.** Baseline Characteristics of Patients Prescribed Thiazide Diuretics
