# Supplementary material for: Association of Hydroxylmethyl Glutaryl Coenzyme A Reductase Inhibitors, L-Type Calcium Channel Antagonists, and Biguanides With Rates of Psychiatric Hospitalization and Self-Harm in Individuals With Serious Mental Illness
Source: JAMA Psychiatry. 2019 Jan 9;76(4):382–90. doi: 10.1001/jamapsychiatry.2018.3907 (PMC6450278; doi:10.1001/jamapsychiatry.2018.3907)
Supplement: Supplement. — eFigure. Patient Flow Diagram eTable 1. ICD Diagnostic Codes eTable 2. Anatomical Therapeutic Chemical Classification System Codes eTable 3. Baseline Characteristics of Patients Treated for Self-Harm eTable 4. Baseline Characteristics of Patients Experiencing Nonpsychiatric Hospitalization eTable 5. Baseline Characteristics of Patients Prescribed Thiazide Diuretics [file jamapsychiatry-76-382-s001.pdf]

Supplementary Online Content

Hayes JF, Lundin A, Wicks S, et al. Association of hydroxylmethyl glutaryl coenzyme A reductase inhibitors, L-type calcium channel antagonists, and biguanides with rates of psychiatric hospitalization and self-harm in individuals with serious mental illness. *JAMA Psychiatry*. Published online January 9, 2019. doi:10.1001/jamapsychiatry.2018.3907

eFigure. Patient Flow Diagram

eTable 1. ICD Diagnostic Codes

eTable 2. Anatomical Therapeutic Chemical Classification System Codes

eTable 3. Baseline Characteristics of Patients Treated for Self-Harm

eTable 4. Baseline Characteristics of Patients Experiencing Nonpsychiatric Hospitalization

eTable 5. Baseline characteristics of Patients Prescribed Thiazide Diuretics

© 2019 Hayes JF et al. *JAMA Psychiatry.*

eFigure. Patient Flow Diagram

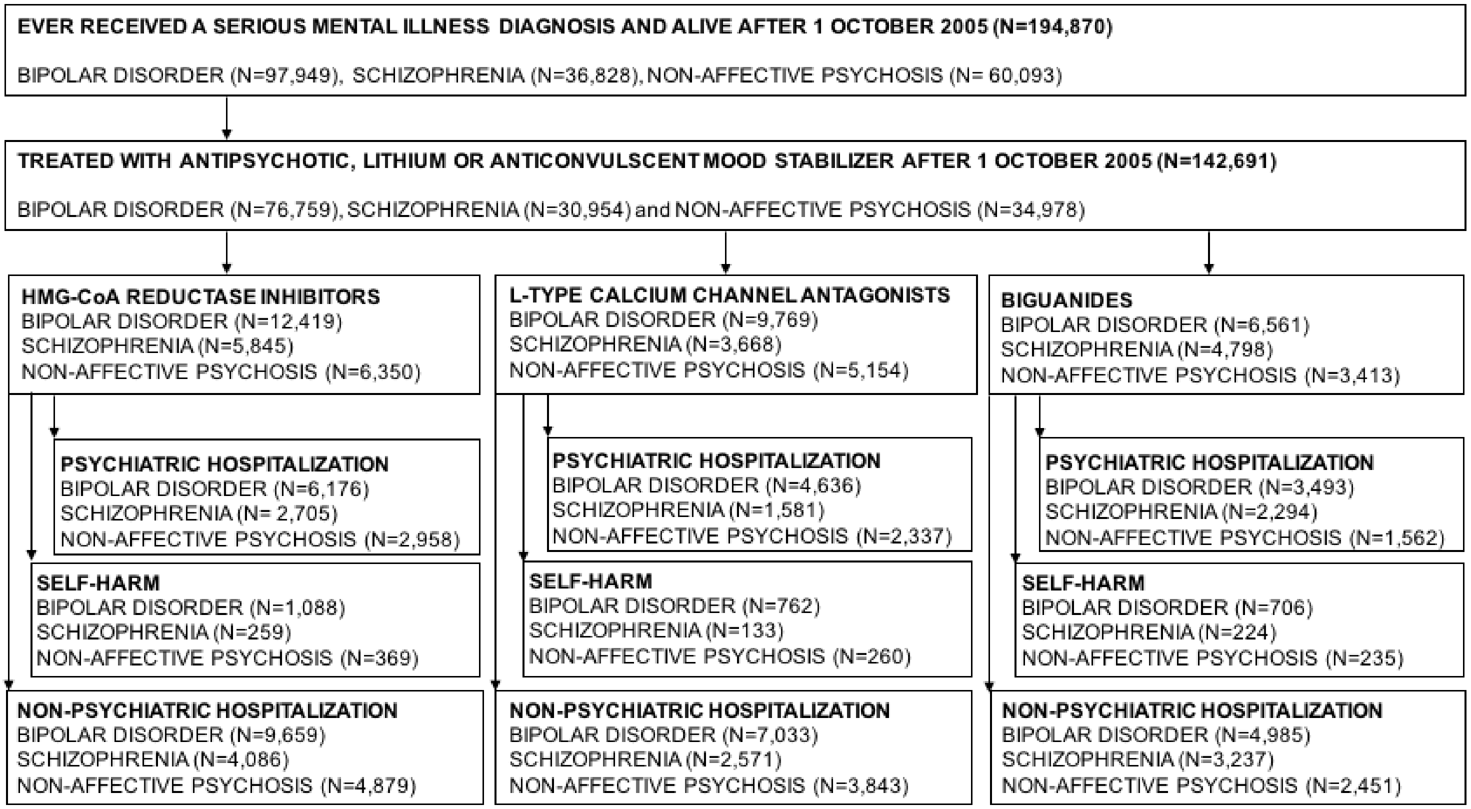

© 2019 Hayes JF et al. *JAMA Psychiatry.*

eTable 1. ***ICD*** **Diagnostic Codes**

**Diagnosis**	***ICD-9***	***ICD-10***
BPD	296A, C-E, W, X	F30-F31n
Schizophrenia	295A-E, G, W, X	F20n
NAP	297, 298B-E, W, X	F21-F24n, F28-F29n
Cardiovascular or cerebrovascular disease	390-405, 410-429, 440-459, 430-438	I00-I15, I20-I52, I60-I99,
Type II diabetes mellitus	250	E10-14
hypertension	401	I10
hyperlipidemia	272	E78
Self-harm	E950-959 (with suicidal intent), E980-989 (undetermined intent)	X60-X84 (with suicidal intent), Y10-Y34 (undetermined intent)

© 2019 Hayes JF et al. *JAMA Psychiatry.*

eTable 2. Anatomical Therapeutic Chemical Classification System Codes

**Drug class**	**Drug name**	**ATC Code**
HMG-CoA reductase inhibitors	Simvastatin	C10AA01
	Lovastatin	C10AA02
	Pravastatin	C10AA03
	Fluvastatin	C10AA04
	Atorvastatin	C10AA05
	Cerivastatin	C10AA06
	Rosuvastatin	C10AA07
	Pitavastatin	C10AA08
LTCC antagonist	Amlodipine	C08CA01
	Felodipine	C08CA02
	Isradipine	C08CA03
	Nicardipine	C08CA04
	Nifedipine	C08CA05
	Nimodipine	C08CA06
	Nisoldipine	C08CA07

© 2019 Hayes JF et al. *JAMA Psychiatry.*

	Nitrendipine	C08CA08
	Lacidipine	C08CA09
	Nilvadipine	C08CA10
	Manidipine	C08CA11
	Barnidipine	C08CA12
	Lercanidipine	C08CA13
	Cilnidipine	C08CA14
	Benidipine	C08CA15
	Clevidipine	C08CA16
	Nifedipine, combinations	C08CA55
	Verapamil	C08DA01
	Gallopamil	C08DA02
	Verapamil, combinations	C08DA51
	Diltiazem	C08DB01
Biguanides	Phenformin	A10BA01
	Metformin	A10BA02
	Buformin	A10BA03

© 2019 Hayes JF et al. *JAMA Psychiatry.*

Thiazide diuretics	Bendroflumethiazide	C03AA01
	Hydroflumethiazide	C03AA02
	Hydrochlorothiazide	C03AA03
	Chlorothiazide	C03AA04
	Polythiazide	C03AA05
	Trichlormethiazide	C03AA06
	Cyclopenthiazide	C03AA07
	Methyclothiazide	C03AA08
	Cyclothiazide	C03AA09
	Mebutizide	C03AA13
Typical antipsychotics	Chlorpromazine	N05AA01
	Levomepromazine	N05AA02
	Promazine	N05AA03
	Acepromazine	N05AA04
	Triflupromazine	N05AA05
	Cyamemazine	N05AA06
	Chlorproethazine	N05AA07

© 2019 Hayes JF et al. *JAMA Psychiatry.*

	Dixyrazine	N05AB01
	Fluphenazine	N05AB02
	Perphenazine	N05AB03
	Prochlorperazine	N05AB04
	Thiopropazate	N05AB05
	Trifluoperazine	N05AB06
	Acetophenazine	N05AB07
	Thioproperazine	N05AB08
	Butaperazine	N05AB09
	Perazine	N05AB10
	Periciazine	N05AC01
	Thioridazine	N05AC02
	Mesoridazine	N05AC03
	Pipotiazine	N05AC04
	Haloperidol	N05AD01
	Trifluperidol	N05AD02
	Melperone	N05AD03

© 2019 Hayes JF et al. *JAMA Psychiatry.*

	Moperone	N05AD04
	Pipamperone	N05AD05
	Bromperidol	N05AD06
	Benperidol	N05AD07
	Droperidol	N05AD08
	Fluanisone	N05AD09
	Flupentixol	N05AF01
	Clopenthixol	N05AF02
	Chlorprothixene	N05AF03
	Thiothixene	N05AF04
	Zuclopenthixol	N05AF05
	Fluspirilene	N05AG01
	Pimozide	N05AG02
	Penfluridol	N05AG03
	Prothipendyl	N05AX07
Atypical antipsychotics	Risperidone	N05AX08
	Mosapramine	N05AX10

© 2019 Hayes JF et al. *JAMA Psychiatry.*

	Zotepine	N05AX11
	Aripiprazole	N05AX12
	Paliperidone	N05AX13
	Iloperidone	N05AX14
	Cariprazine	N05AX15
	Brexpiprazole	N05AX16
	Oxypertine	N05AE01
	Molindone	N05AE02
	Sertindole	N05AE03
	Ziprasidone	N05AE04
	Lurasidone	N05AE05
	Loxapine	N05AH01
	Clozapine	N05AH02
	Olanzapine	N05AH03
	Quetiapine	N05AH04
	Asenapine	N05AH05
	Clotiapine	N05AH06

© 2019 Hayes JF et al. *JAMA Psychiatry.*

	Sulpiride	N05AL01
	Sultopride	N05AL02
	Tiapride	N05AL03
	Remoxipride	N05AL04
	Amisulpride	N05AL05
	Veralipride	N05AL06
	Levosulpiride	N05AL07
Lithium	Lithium	N05AN01
Anticonvulsant class mood stabilizers	Valproic acid	N03AG01
	Lamotrigine	N03AX09
	Carbamazepine	N03AF01

© 2019 Hayes JF et al. *JAMA Psychiatry.*

eTable 3. Baseline Characteristics of Patients Treated for Self-Harm

	**HMG-CoA reductase inhibitors**			**L-type calcium channel antagonists**			**Biguanides**		
	BPD	Schizophrenia	NAP	BPD	Schizophrenia	NAP	BPD	Schizophrenia	NAP
**N**	1,088	259	369	762	133	260	706	224	235
**Age at start of follow-up, mean (SD)**	50.27 (12.76)	44.52 (13.39)	49.66 (15.92)	51.17 (13.93)	50.15 (12.97)	52.73 (16.13)	43.39 (15.09)	42.44 (13.55)	42.95 (15.49)
**Female sex, N (%)**	665 (61.12)	129 (49.81)	194 (52.57)	460 (60.37)	64 (48.12)	137 (52.69)	450 (63.74)	120 (53.57)	131 (55.74)
**Swedish born, N (%)**	915 (84.10)	192 (74.13)	289 (78.32)	646 (84.78)	105 (78.95)	198 (76.15)	579 (82.01)	159 (70.98)	181 (77.02)
**Died during follow-up, N (%)**	179 (16.45)	36 (13.90)	65 (17.62)	153 (20.08)	37 (27.82)	62 (23.85)	111 (15.72)	39 (17.41)	32 (13.62)
**Psychiatric treatment during follow-up, N (%):**									
Antipsychotic	940 (86.40)	255 (98.46)	351 (95.12)	621 (81.50)	133 (100)	240 (92.31)	648 (91.78)	224 (100)	223 (94.89)
Lithium	504 (46.32)	25 (9.65)	21 (5.69)	342 (44.88)	5 (3.76)	10 (3.85)	357 (50.57)	19 (8.48)	20 (8.51)
Anticonvulsant	759 (69.76)	79 (30.50)	105 (28.46)	497 (65.22)	42 (31.58)	61 (23.46)	499 (70.68)	70 (31.25)	86 (36.60)
**Physical health by end of follow-up, N (%):**									
Cardiovascular or cerebrovascular disease	378 (34.74)	66 (25.48)	133 (36.04)	639 (83.86)	105 (78.95)	225 (86.54)	417 (59.07)	120 (53.57)	142 (60.43)
Type II Diabetes Mellitus	173 (15.90)	55 (21.24)	53 (14.36)	189 (24.80)	54 (40.60)	75 (28.85)	488 (69.12)	166 (74.11)	166 (70.64)
Hypertension	198 (18.20)	30 (11.58)	58 (15.72)	570 (74.80)	92 (69.17)	197 (75.77)	296 (41.93)	73 (32.59)	103 (43.83)

© 2019 Hayes JF et al. *JAMA Psychiatry.*

Hyperlipidemia	82 (7.54)	14 (5.41)	35 (9.49)	90 (11.81)	12 (9.02)	30 (11.54)	99 (14.02)	25 (11.16)	27 (11.49)
**Number of study drug exposure periods, median (IQR)**	5 (3-9)	3 (3-7)	5 (3-9)	5 (3-9)	3 (3-5)	5 (3-9)	3 (3-7)	4 (3-7)	3 (3-7)
**Rate of admission per 100 person-years (95% CI)**	23.81 (22.91-24.75)	24.46 (22.63-26.45)	23.94 (22.39-25.59)	20.27 (19.28-21.31)	17.52 (15.35-20.01)	19.14 (17.51-20.92)	27.47 (26.27-28.72)	22.14 (20.43-24.37)	29.62 (27.49-31.92)
**Psychiatric admissions on/off study drug, N**	587/ 1984	130/ 499	240/ 623	292/ 1242	41/ 177	112/ 374	491/ 1429	151/ 343	166/ 521
**Time on/off study drug, person-years**	$2.6x10^3$/ $8.2x10^3$	$7.8x10^2$/ $1.8x10^3$	$8.6x10^2$/ $2.7x10^3$	$1.5x10^3$/ $6.1x10^3$	$3.1x10^2$/ $9.3x10^2$	$5.6x10^2$/ $2.0x10^3$	$2.1x10^3$/ $4.9x10^3$	$8.1x10^2$/ $1.4x10^3$	$6.5x10^2$/ $1.7x10^3$

© 2019 Hayes JF et al. *JAMA Psychiatry.*

eTable 4. Baseline Characteristics of Patients Experiencing Nonpsychiatric Hospitalization

	HMG-CoA reductase inhibitors			L-type calcium channel antagonists			Biguanides		
	BPD	Schizophrenia	NAP	BPD	Schizophrenia	NAP	BPD	Schizophrenia	NAP
**N**	9,659	4,086	4,879	7,033	2,571	3,843	4,985	3,237	2451
**Age at start of follow-up, mean (SD)**	57.34 (12.51)	54.53 (12.56)	60.98 (14.29)	58.29 (13.63)	58.65 (12.31)	64.60 (14.91)	53.29 (14.72)	53.27 (13.44)	55.78 (15.98)
**Female sex, N (%)**	5,483 (56.77)	1,794 (43.91)	2,790 (57.18)	4,147 (58.96)	1,222 (47.53)	2,442 (63.69)	2,943 (59.04)	1,464 (45.23)	1,392 (56.79)
**Swedish born, N (%)**	8,331 (86.25)	3,163 (77.41)	3,705 (75.94)	6,119 (87.00)	2,005 (77.99)	3,010 (78.51)	4,195 (84.15)	2,439 (75.35)	1,701 (69.40)
**Died during follow-up, N (%)**	2,250 (23.29)	1,113 (27.24)	1,551 (31.79)	1,816 (25.82)	943 (36.68)	1,472 (38.39)	1,164 (23.35)	949 (29.32)	694 (28.31)
**Psychiatric treatment during follow-up, N (%):**									
Antipsychotic	7,451 (77.14)	4,046 (99.02)	4,656 (95.43)	5,193 (73.84)	2,541 (98.83)	3,658 (95.41)	4,161 (83.47)	3,223 (99.57)	2,372 (96.78)
Lithium	4,363 (45.17)	133 (3.26)	110 (2.25)	3,250 (46.21)	72 (2.80)	66 (1.72)	2,134 (42.81)	117 (3.61)	83 (3.39)
Anticonvulsant	4,983 (51.59)	700 (17.13)	762 (15.62)	3,627 (51.57)	406 (15.79)	538 (14.03)	2,599 (52.14)	532 (16.43)	387 (15.79)
**Physical health by end of follow-up, N (%):**									
Cardiovascular or cerebrovascular disease	7,748 (80.22)	3,024 (74.01)	4,092 (83.87)	6,131 (87.17)	2,240 (87.13)	3,488 (90.98)	3,466 (69.53)	2,096 (64.75)	1,815 (74.05)
Type II Diabetes Mellitus	3,802 (39.36)	2,156 (52.77)	1,919 (39.33)	2,006 (28.52)	1,010 (39.28)	1,118 (29.16)	3,985 (79.94)	2,711 (83.75)	2,028 (82.74)
Hypertension	5,482 (56.76)	1,934 (47.33)	3,070 (62.92)	5,414 (76.98)	1,939 (75.42)	3,100 (80.86)	2,585 (51.86)	1,394 (43.06)	1,384 (56.47)
Hyperlipidemia	2,001	676	1,143	878	242	554	739	370	363

© 2019 Hayes JF et al. *JAMA Psychiatry.*

	(20.72)	(16.54)	(23.43)	(12.48)	(9.41)	(14.45)	(14.82)	(11.43)	(14.81)
**Number of study drug exposure periods, mean (SD)**	5 (2-10)	4 (2-8)	5 (2-9)	5 (3-11)	3 (3-7)	5 (3-11)	4 (3-7)	3 (3-7)	5 (3-9)
**Rate of admission per 100 person-years (95% CI)**	52.69 (52.23-53.15)	45.74 (45.08-46.40)	58.86 (58.17-59.55)	47.57 (47.35-47.79)	46.85 (46.49-47.23)	61.32 (60.92-61.72)	51.73 (51.10-52.37)	42.12 (41.41-42.84)	53.35 (52.42-54.28)
**Psychiatric admissions on/off study drug, N**	14865/ 36470	6838/ 11684	7929/ 20131	8918/ 28459	4131/ 8426	5325/ 17301	6390/ 19349	4811/ 8531	3053/ 9546
**Time on/off study drug, person-years**	$2.3x10^{4}$/ $7.4x10^{4}$	$1.3x10^{4}$/ $2.7x10^{4}$	$1.2x10^{4}$/ $3.6x10^{4}$	$1.5x10^{4}$/ $5.7x10^{4}$	$7.3x10^{3}$/ $1.8x10^{4}$	$8.1x10^{3}$/ $2.9.4x10^{4}$	$1.3x10^{4}$/ $3.7x10^{4}$	$1.1x10^{4}$/ $2.0x10^{4}$	$5.9x10^{3}$/ $1.8x10^{4}$

© 2019 Hayes JF et al. *JAMA Psychiatry.*

eTable 5. Baseline Characteristics of Patients Prescribed Thiazide Diuretics

	Psychiatric hospitalization	Self-harm	Nonpsychiatric hospitalization
**N**	2,685	365	4,309
**Age at start of follow-up, mean (SD)**	58.78 (13.60)	52.87 (13.15)	61.46 (13.76)
**Female sex, N (%)**	1,692 (63.02)	229 (62.74)	2,757 (63.98)
**Swedish born, N (%)**	2,274 (85.10)	303 (83.01)	3,677 (85.33)
**Died during follow-up, N (%)**	705 (26.26)	80 (21.92)	1,365 (31.68)
**Psychiatric treatment during follow-up, N (%):**			
Antipsychotic	2,475 (92.18)	322 (88.22)	3,725 (86.45)
Lithium	649 (24.17)	94 (25.75)	909 (21.10)
Anticonvulsant	1,043 (38.85)	202 (55.34)	1,486 (34.49)
**Physical health by end of follow-up, N (%):**			
Cardiovascular or cerebrovascular disease	2,212 (82.38)	307 (84.11)	3,787 (87.89)
Type II Diabetes Mellitus	739 (27.52)	97 (26.58)	1,280 (29.71)
Hypertension	1,951 (72.66)	272 (74.52)	3,343 (77.58)
Hyperlipidemia	305 (11.36)	44 (12.05)	512 (11.88)
**Number of study drug exposure periods, median (IQR)**	5 (3-9)	3 (3-7)	3 (3-7)
**Rate of admission per 100 person-years (95% CI)**	41.51 (40.76-42.28)	23.47 (21.88-25.18)	51.72 (51.03-52.41)
**Psychiatric admissions on/off study drug, N**	1755/9674	151/626	3452/18369
**Time on/off study drug, person-years**	$4.6x10^3/2.3x10^4$	$5.9x10^2/2.7x10^3$	$7.1x10^3/3.5x10^4$

© 2019 Hayes JF et al. *JAMA Psychiatry.*
